# Nrf2 status affects tumor growth, HDAC3 gene promoter associations, and the response to sulforaphane in the colon

**DOI:** 10.1186/s13148-015-0132-y

**Published:** 2015-09-18

**Authors:** Praveen Rajendran, Wan-Mohaiza Dashwood, Li Li, Yuki Kang, Eunah Kim, Gavin Johnson, Kay A. Fischer, Christiane V. Löhr, David E. Williams, Emily Ho, Masayuki Yamamoto, David A. Lieberman, Roderick H. Dashwood

**Affiliations:** Center for Epigenetics & Disease Prevention, Texas A&M Health Science Center, Houston, TX USA; Linus Pauling Institute, Oregon State University, Corvallis, OR USA; College of Veterinary Medicine, Oregon State University, Corvallis, OR USA; Department of Environmental and Molecular Toxicology, Oregon State University, Corvallis, OR USA; College of Public Health and Human Sciences, Oregon State University, Corvallis, OR USA; Division of Medical Biochemistry, Tohoku University Graduate School of Medicine, Sendai, Miyagi Japan; Department of Medicine, Oregon Health & Science University, Portland, OR USA; Department of Food Science & Nutrition, Texas A&M University, College Station, TX USA; Department of Molecular & Cellular Medicine, Texas A&M University, College Station, TX USA; Department of Clinical Cancer Prevention, MD Anderson Cancer Center, Houston, TX USA

**Keywords:** HDAC3, *p16*, Nrf2, Colon cancer, Sulforaphane, Broccoli

## Abstract

**Background:**

The dietary agent sulforaphane (SFN) has been reported to induce nuclear factor erythroid 2 (NF-E2)-related factor 2 (Nrf2)-dependent pathways as well as inhibiting histone deacetylase (HDAC) activity. The current investigation sought to examine the relationships between Nrf2 status and HDAC expression in preclinical and translational studies.

**Results:**

Wild type (WT) and Nrf2-deficient (Nrf2^−/+^) mice were treated with the colon carcinogen 1,2-dimethylhydrazine (DMH) followed by 400 ppm SFN in the diet (*n* = 35 mice/group). WT mice were more susceptible than Nrf2^−/+^ mice to tumor induction in the colon. Tumors from WT mice had higher HDAC levels globally and locally on genes such as *cyclin-dependant kinase inhibitor 2a (Cdkn2a/p16)* that were dysregulated during tumor development. The average tumor burden was reduced by SFN from 62.7 to 26.0 mm^3^ in WT mice and from 14.6 to 11.7 mm^3^ in Nrf2^−/+^ mice. The decreased antitumor activity of SFN in Nrf2^−/+^ mice coincided with attenuated *Cdkn2a* promoter interactions involving HDAC3. HDAC3 knockdown in human colon cancer cells recapitulated the effects of SFN on p16 induction. Human subjects given a broccoli sprout extract supplement (200 μmol SFN equivalents), or reporting more than five cruciferous vegetable servings per week, had increased *p16* expression that was inversely associated with HDAC3 in circulating peripheral blood mononuclear cells (PBMCs) and in biopsies obtained during screening colonoscopy.

**Conclusions:**

Nrf2 expression varies widely in both normal human colon and human colon cancers and likely contributes to the overall rate of tumor growth in the large intestine. It remains to be determined whether this influences global HDAC protein expression levels, as well as local HDAC interactions on genes dysregulated during human colon tumor development. If corroborated in future studies, Nrf2 status might serve as a biomarker of HDAC inhibitor efficacy in clinical trials using single agent or combination modalities to slow, halt, or regress the progression to later stages of solid tumors and hematological malignancies.

**Electronic supplementary material:**

The online version of this article (doi:10.1186/s13148-015-0132-y) contains supplementary material, which is available to authorized users.

## Background

Histone deacetylase (HDAC) enzymes have emerged as important regulators of cancer development [1, 2]. Downregulation of specific HDACs can increase global histone acetylation, turn-on epigenetically silenced genes, and trigger cell cycle arrest, apoptosis, or differentiation in cancer cells [3–5]. Pan-HDAC inhibitors are currently undergoing clinical evaluation as anticancer agents, but the quest continues for more specific HDAC inhibitors with improved efficacy towards hematological and solid tumors [5].

We reported that a natural compound, sulforaphane (SFN), targets HDAC3 for protein turnover in human colon cancer cells [6–12]. SFN is obtained from broccoli and other cruciferous vegetables that are rich sources of the precursor, glucoraphanin [13, 14]. SFN was first identified as an inducer of phase 2 detoxification enzymes, acting via the nuclear factor erythroid 2 (NF-E2)-related factor 2 (Nrf2) signaling pathway [15]. However, Nrf2 in certain circumstances can preserve rather than attenuate cancer phenotypes [16, 17].

The current investigation sought to examine the relationships between Nrf2 status and HDAC expression in a widely used model of colon carcinogenesis [18, 19]. Preclinical experiments included post-initiation SFN treatment and highlighted a role for HDAC3 in regulating cyclin-dependant kinase inhibitor 2a (*Cdkn2a*/*p16)* expression. The findings were extended to human subjects according to their cruciferous vegetable consumption or supplement intake.

## Results

### Dosing schedule and HDAC3 levels dictate anticancer outcomes in the colon

**Fig. 1:**
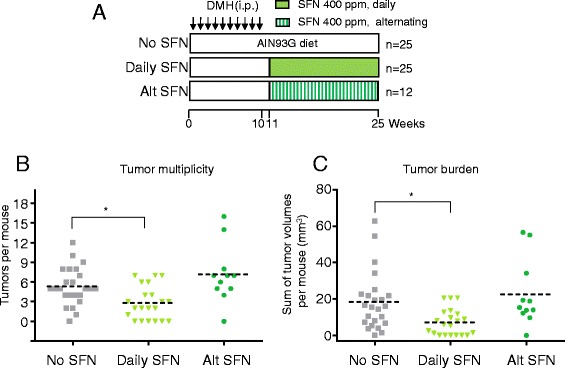
Continuous and alternating dosing schedules for dietary SFN in the DMH model. **a** Male ICR mice were injected *i.p.* with DMH (20 mg/kg), once per week for 10 weeks (*arrows*). One week after completing the DMH treatment, mice were continued on standard AIN93 diet (no SFN) or given AIN93 diet containing 400 ppm SFN either continuously (daily SFN, *green box*) or on alternate days (Alt SFN, *stripes*). **b** Tumor multiplicity and **c** total tumor burden, i.e., the sum of individual tumor volumes, were determined for each animal at 25 weeks, and mean values were calculated (*dotted line*). *P < 0.05 vs. no SFN controls

**Fig. 2:**
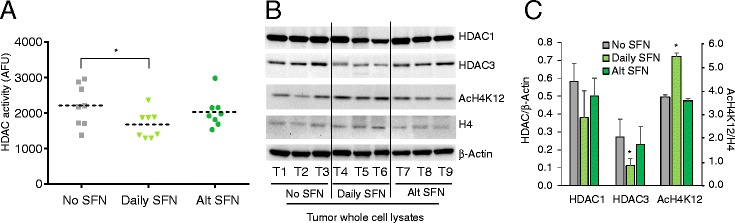
SFN decreased HDAC activity and HDAC3 protein expression in mouse colon tumors. **a** HDAC activity in colon tumor lysates was determined as described in “Methods” section. Mean values were calculated in each group (*dotted line*). *P < 0.05 vs. no SFN controls. **b** HDAC expression and histone acetylation determined by immunoblotting. **c** Densitometry data for HDACs were normalized to β-actin, whereas acetylated histone H4K12 (AcH4K12) was normalized to histone H4. Data = mean ± SD (*n* = 3); *P < 0.05 vs. no SFN controls

1,2-Dimethylhydrazine (DMH) was administered for 10 weeks, and 1 week later SFN was given in the diet by continuous or alternating daily dosing schedules (Fig. 1a). There was a significant reduction in tumor multiplicity and tumor burden only after continuous SFN treatment (Fig. 1b, c). In colon tumors, daily SFN lowered HDAC activity (Fig. 2a) and HDAC3 protein expression (Fig. 2b, c) while increasing global histone H4 acetylation (Fig. 2b, c). The findings in vivo recapitulate prior observations on HDAC3 protein turnover by SFN in cell-based assays [20]. Several SFN metabolites have been examined in the context of the HDAC turnover mechanism [11, 20, 21], namely, SFN-glutathione (SFN-GSH), SFN-cysteine-glycine (SFN-CG), SFN-cysteine (SFN-Cys), and SFN-*n*-acetylcysteine (SFN-NAC). These metabolites were detected, as reported [22], in tissues of SFN-treated mice (data not shown).

### Nrf2 genetic background influences HDAC protein levels in colon tumors

**Fig. 3:**
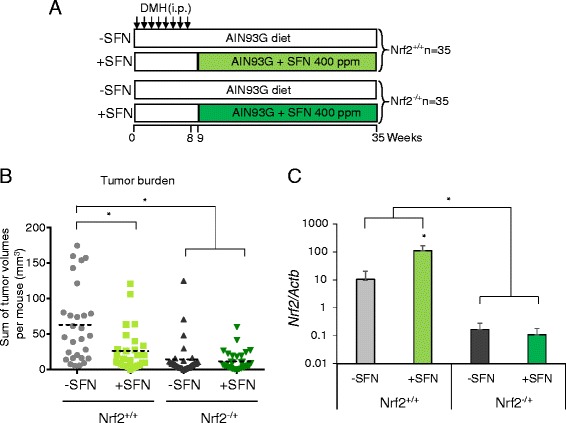
Antitumor activity of dietary SFN in Nrf2^+/+^ and Nrf2^−/+^ mice. **a** Nrf2^+/+^ (WT) or Nrf2^−/+^ mice were injected *i.p.* with DMH (20 mg/kg), once per week for 8 weeks (*arrows*). One week after completing the carcinogen treatment, mice were continued on standard AIN93 diet (−SFN) or AIN93 diet containing 400 ppm SFN (+SFN). **b** Total tumor burden/mouse indicates the sum of individual tumor volumes after 35 weeks, plotted for each animal; *dotted line* represents the mean value in each group; *P* < 0.05 as indicated by the *asterisk*. **c** Relative mRNA expression levels of *Nrf2* normalized to the β-actin gene, *Actb*. Data = mean ± SD (*n* = 3)

Continuous SFN treatment was used in a modified protocol that employed fewer DMH doses and a longer post-initiation phase in wild type (WT, Nrf2^+/+^) and Nrf2^−/+^ mice (Fig. 3a). The longer study duration of 35 weeks resulted in an average tumor burden of 62.7 mm^3^ in WT mice (Fig. 3b and Table 1, part B), compared with 18.4 mm^3^ at 25 weeks (Table 1, part A). Notably, in the 35-week study, DMH produced an average tumor burden of 14.6 mm^3^ in Nrf2^−/+^ mice (Fig. 3b and Table 1, part B), compared with 62.7 mm^3^ in the Nrf2^+/+^ controls. In SFN-fed mice, the average tumor burden was reduced from 62.7 to 26.0 mm^3^ in WT mice and from 14.6 to 11.7 mm^3^ in Nrf2^−/+^ mice (Fig. 3b and Table 1, part B). In colon tumors, *Nrf2* levels were significantly lower in Nrf2^−/+^ vs. Nrf2^+/+^ mice, regardless of SFN treatment (Fig. 3c).

**Table 1:** Summary of mouse tumor studies

Treatment	Colon tumor incidence	Multiplicity (tumors/mouse)	Tumor burden (mm^3^)	Average food consumption (g/day)	Mouse weight at end of study (g)
A 25-week study					
No SFN	23/24 (95.8 %)	5.3 ± 2.8	18.4 ± 15.9	3.5 ± 0.8	45.3 ± 4.9
Daily SFN	15/21 (71.4 %)	2.8 ± 2.5*	7.1 ± 7.2*	3.9 ± 1.3	43.6 ± 3.8
Alt SFN	10/11 (90.9 %)	7.2 ± 4.4	22.6 ± 18.3	3.8 ± 1.0	44.0 ± 2.9
B 35-week study					
−SFN (Nrf2^+/+^)	28/28 (100 %)	7.0 ± 3.7	62.7 ± 53.5	3.7 ± 0.7	51.3 ± 6.5
+SFN (Nrf2^+/+^)	27/28 (96.4 %)	5.7 ± 4.0	26.0 ± 30.2*	3.6 ± 1.5	44.7 ± 7.0
−SFN (Nrf2^−/+^)	31/32 (96.8 %)	4.0 ± 2.1	14.6 ± 24.8	3.1 ± 0.6	45.2 ± 8.0
+SFN (Nrf2^−/+^)	28/31 (90.3 %)	3.0 ± 2.1	11.7 ± 13.5	3.2 ± 0.5	43.8 ± 6.5

*P < 0.05 vs. corresponding control mice given no dietary SFN

**Fig. 4:**
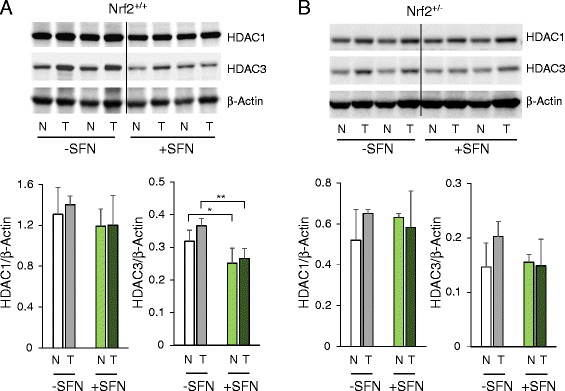
SFN decreased HDAC3 protein expression in colon tumors of Nrf2^+/+^ but not Nrf2^−/+^ mice. **a** HDAC expression was immunoblotted in tumor (*T*) and adjacent normal-looking colon (*N*) of WT mice, with densitometry data normalized to β-actin. **b** The corresponding data for Nrf2^−/+^ mice. Data are given as mean ± SD (*n* = 3); *P < 0.05, **P < 0.01 for control diet vs. SFN treatment. In addition to global reductions in HDAC3, marked loss of HDAC3 on gene targets was examined (see Fig. 6)

Immunoblotting of tissue lysates from tumor and adjacent normal colon revealed that HDAC3 expression was reduced by SFN treatment in WT mice (Fig. 4a), but not in mice on the Nrf2^−/+^ background (Fig. 4b). In general, HDAC expression was lower in colon tissues of Nrf2^−/+^ animals (compare *y*-axes of densitometry plots in Fig. 4a, b). No treatment-related differences were detected for *HDAC3* mRNA levels or for other class I *HDAC*s (data not shown).

### Nrf2 status impacts HDAC3 levels on *p16* in mouse colon tumors

**Fig. 5:**
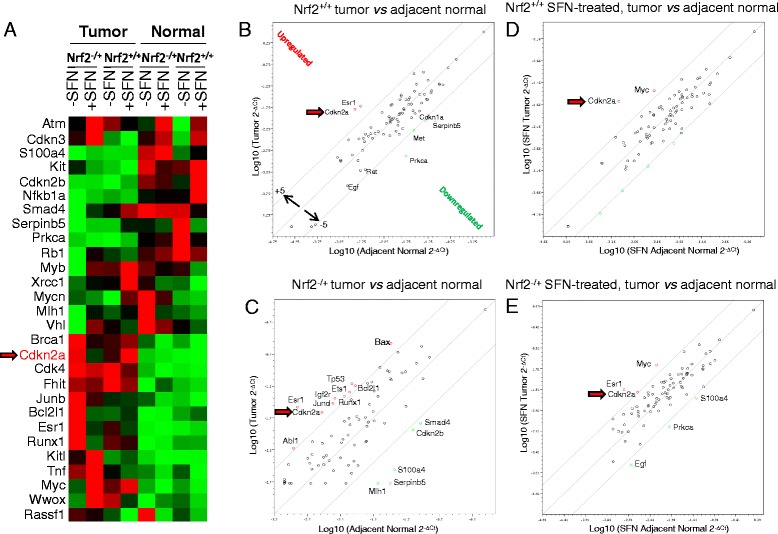
Differential gene expression in mouse colon tumors and normal colon. **a** Heat map representing the expression of differentially expressed genes, relative to *Actb*. Each *vertical column* represents the gene expression profile of pooled colon tumors or adjacent normal colon (*n* = 6) of mice on standard AIN93 diet (−SFN) or AIN93 diet containing 400 ppm SFN (+SFN), according to the groups indicated. *Each row* in the heat map represents a single named gene. *Red* represents high expression, *green* designates low expression. **b**–**e** Scatter plots compared the normalized expression of genes in the array by plotting log_10_ transformed 2^−Δ*C*t^ values between selected groups. The most significantly altered genes, with an arbitrary cutoff of fivefold in either direction (upregulated or downregulated), are identified by the name adjacent to the corresponding data point. The *red arrow* designates *Cdkn2a* (*p16*)

Gene expression arrays differentiated between the genes most altered by SFN treatment when comparing tumor with adjacent normal colon (Fig. 5a). For the complete list of genes and the respective fold changes, see Additional file 1: Table S1. Scatter plots were generated with an arbitrary cutoff of fivefold in either direction. When tumors were compared with adjacent normal colon, *cyclin-dependant kinase inhibitor 2A (Cdkn2a/P16),* a well-known tumor suppressor, was surprisingly the most highly overexpressed gene in the tumors of WT mice (Fig. 5b). Estrogen receptor 1, alpha (*Esr1*), a nuclear hormone receptor involved in the regulation of eukaryotic gene expression that affects cellular proliferation and differentiation in target tissues, also was highly expressed, whereas known tumor suppressors cyclin-dependant kinase 1A (*Cdkn1a/p21*), serine peptidase inhibitor (s*erpin b5*), and oncogenes such as Met proto-oncogene (*Met*), and protein kinase C, alpha (*Prkca*) were underexpressed in the tumor. On the Nrf2^−/+^ background (Fig. 5c), tumor suppressor genes such as *Cdkn2a* and transformation-related protein 53 (*Tp53*), as well as reported oncogenic factors E26 avian leukemia oncogene 1,5′ domain (*Ets1*) and insulin-like growth factor 2 receptor (*Igf2r*) were overexpressed, whereas underexpressed genes included tumor suppressor genes cyclin-dependent kinase inhibitor 2B (*Cdkn2b/p15*) and *serpin b5*. mutL homolog 1 (*Mlh1*), which is known to be silenced in human colon cancer was also attenuated in mouse colon tumors. SFN treatment compressed the spread of altered genes (Fig. 5d, e), although *Cdkn2a/p16* levels remained consistently elevated, especially in WT mice given SFN.

**Fig. 6:**
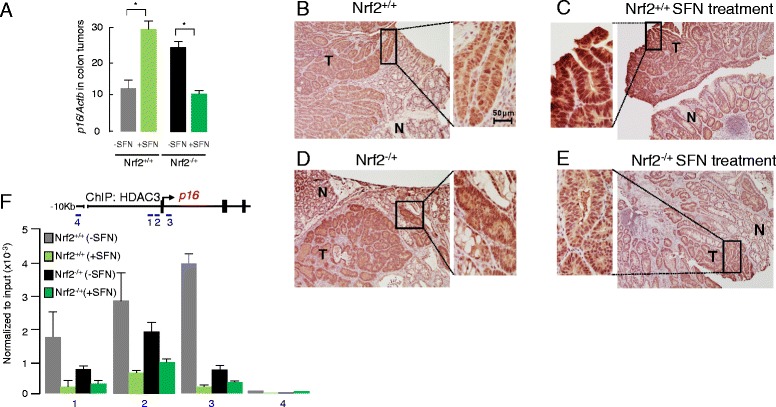
Nrf2 status affects *p16* expression and HDAC3 promoter interactions in mouse colon tumors. **a** *p16* mRNA expression levels in mouse colon tumors were normalized to *Actb*. Data = mean ± SD (*n* = 3); *P < 0.05 for control diet vs. SFN treatment. **b**–**e** Representative immunohistochemical staining (×10) of p16 in mouse colon tumor and adjacent normal colon; tumor (*T*), normal (*N*). A magnified (×40) image is shown for each *dotted box*, with a 50-μm scale bar included in **b**, *inset*. **f** In vivo ChIP assays on mouse colon tumors, interrogating HDAC3 interactions on *p16.* Data = mean ± SD and are representative of three independent experiments. A region 10 Kb upstream of the *p16* promoter, containing a Runx1 binding site, served as a negative control for the ChIP assays

Candidates from the PCR arrays were validated by quantitative reverse transcription PCR (qRT-PCR), confirming *Cdkn2a/p16* as being among the most highly overexpressed genes (Additional file 2: Figure S1). SFN altered *Cdkn2a/p16* mRNA expression in colon tumor and adjacent normal-looking colon of WT and Nrf2^−/+^ mice (compare red arrows in Additional file 2: Figure S1, A vs. B, C vs. D, E vs. F, and G vs. H). Interestingly, SFN increased *Cdkn2a/p16* mRNA levels in colon tumors of WT mice but had the opposite effect in Nrf2^−/+^ mice (Fig. 6a). These findings were supported by immunohistochemical analysis of p16 protein expression (Fig. 6b–e). Thus, high levels of p16 protein were detected in tumors compared with adjacent normal colon, especially in WT mice fed with SFN (Fig. 6c, inset).

Based on prior reports linking *Cdkn2a* and HDAC3 [23–25], we performed chromatin immunoprecipitation (ChIP) assays in vivo and observed HDAC3 interactions on the *p16* proximal promoter region to be lower in colon tumors of Nrf2^−/+^ mice compared with WT (Fig. 6f, black vs. grey bars), especially after SFN treatment (green bars). A runt-related transcription factor 1 (Runx1) binding site, ~10 Kb upstream of the *p16* promoter [26], had minimal HDAC3 interactions and served as a negative control for the ChIP assays (Fig. 6f, region “4”). Notably, the findings for HDAC3-dependent regulation of *p16* did not represent a generic response of all highly dysregulated genes; HDAC3 interactions on *Esr1*, for example, were unaffected by SFN (Additional file 2: Figure S2).

### *p16* is regulated directly by HDAC3, but not Nrf2, in human colon cancer cells

**Fig. 7:**
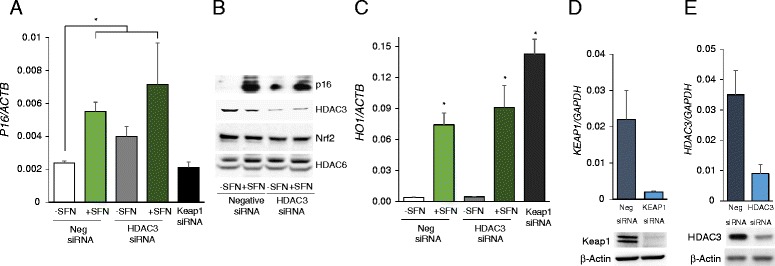
HDAC3 knockdown induces p16 expression in human colon cancer cells. **a** HCT116 cells were transfected with non-specific scrambled negative control siRNA (*Neg siRNA*), HDAC3 siRNA, or Keap1 siRNA for 48 h, followed by vehicle (−SFN) or 15-μM SFN treatment for 24 h. *p16* mRNA levels were normalized to *ACTB;* data = mean ± SD (*n* = 3), *P < 0.05 compared to untreated Neg siRNA. **b** The corresponding whole cell lysates were immunoblotted for p16, HDAC3, HDAC6 and Nrf2. HDAC6 was unchanged and served as loading control. **c** The same samples were analyzed for changes in *heme oxygenase-1* (*HO1*), a known Nrf2 target gene. **d**–**e** Confirmation of Keap1 and HDAC3 knockdown. Kelch-like ECH-associated protein 1 (*Keap1*)

In human colon cancer cells, *Cdkn2a* mRNA expression was induced 24 h after SFN treatment (Fig. 7a), and immunoblotting corroborated the increased expression of p16 protein (Fig. 7b). RNAi-mediated knockdown of HDAC3 also increased *p16* levels, whereas the knockdown of Kelch-like ECH-associated protein 1 (Keap1), which releases Nrf2 from the cytoplasm to the nucleus for gene activation, did not increase *p16* expression. Keap1 knockdown induced the Nrf2 target gene *heme oxygenase-1* (*HO1*, a positive control for Nrf2 activation) to a similar extent as SFN treatment, whereas HDAC3 knockdown had no effect on *HO1* (Fig. 7c). Knockdown of Keap1 and HDAC3 was confirmed at the mRNA and protein level (Fig. 7d, e). We conclude that *Cdkn2a/p16* is an HDAC3-regulated gene that does not involve direct Nrf2 interactions, whereas the reverse is true for *HO1*.

### HDAC3 and p16 are reciprocally regulated in humans after SFN intake

**Fig. 8:**
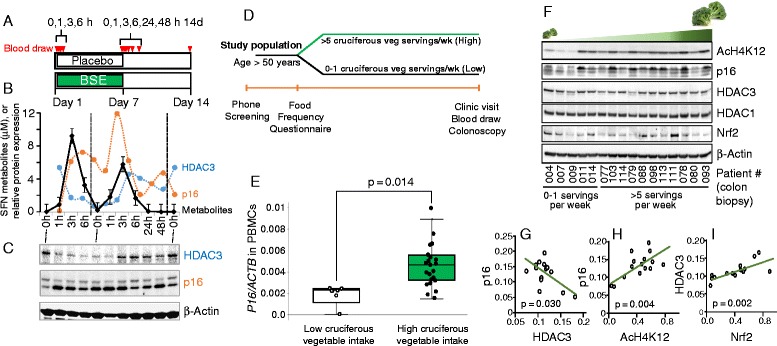
Human subjects given a broccoli sprout extract, or consuming high levels of cruciferous vegetables, have altered HDAC3 and *p16* expression. **a** Human subjects (*n* = 5, each arm of the study) consumed a broccoli sprout extract supplement (BSE, 200 μmol SFN equivalent, IND #111736) or a placebo for 7 days, and blood was drawn at the times indicated. **b** Total levels of SFN and its metabolites in plasma (*black symbols*), and **b**, **c** HDAC3 and p16 protein expression changes in circulating PBMCs. Results are from a single volunteer and are representative of other subjects who took the BSE supplement. No such changes occurred after taking the placebo (data not shown). Individual SFN metabolites, noted in Additional file 3: Table S2, were determined as reported [27]. **d** Study design in screening colonoscopy patients. **e** Relative mRNA expression of *p16* in subjects reporting low (0–1 servings/week) vs. high (>5 servings/week) cruciferous vegetable intake. **f** Immunoblotting of colon biopsies, arranged left to right according to cruciferous vegetable intake. **g**–**i** Associations between p16, HDAC3, AcH4K12, and Nrf2 normalized to β-actin (relative densitometry of proteins in panel **f**)

To examine these relationships in humans, healthy subjects consumed a broccoli sprout extract (BSE) supplement or placebo once a day for 7 days, and blood was drawn at 0, 1, 3, and 6 h post-consumption on day 1 (first day of study) and day 7 (to study effects due to dose accumulation, if any), and on days 8, 9, and 14 as a follow-up to ensure systemic clearance of SFN and metabolites (Fig. 8a). After consuming BSE (200 μmol SFN equivalents), plasma levels of SFN metabolites peaked at 1–3 h, rapidly decreased at 6 h, and were undetectable at 24 h (Fig. 8b, black symbols). The specific SFN metabolites were in agreement with prior studies [27] and were higher on day 1 as compared to day 7 (Additional file 3: Table S2). In a recent report [27], half the dose (100 μmol SFN equivalents) given every 12 h produced lower SFN tissue concentrations than in the present investigation. We infer that a single daily dose of 200 μmol SFN equivalents might be necessary to elicit HDAC inhibitory responses in vivo.

In mice, a single oral dose of SFN reduced HDAC3 protein expression and increased p16 protein level in splenocytes, supporting the use of these end points as potential biomarkers in systemic tissues (Additional file 2: Figure S3). Thus, HDAC3 and p16 protein expression changes also were examined in circulating peripheral blood mononuclear cells (PBMCs) from human volunteers (Fig. 8c). HDAC3 was reduced as early as 1 h after BSE consumption and continued to remain low up to 6 h later. Notably, the loss of HDAC3 coincided with increased p16 expression during this time period. On day 7, HDAC3 levels returned to baseline, whereas p16 remained elevated (Fig. 8c). This implies that cross-talk between HDAC3 and p16 can be uncoupled at later times, possibly via interactions of p16 with alternative HDACs, histone acetyltransferases, and/or their co-regulators [20, 21]. For example, HDAC3/SMRT inhibition and turnover has been observed to precede changes in other HDACs, such as HDAC6 [20]. Lower SFN metabolite levels at day 7 vs. day 1 hinted at a possible compensatory mechanism following repeated daily SFN intake. Further studies are needed to examine the possible induction of pathways that favor enhanced SFN metabolism and/or excretion.

Based on findings from the short-term intervention trial with BSE, HDAC3 and p16 were examined in the context of more typical human dietary intake patterns (Fig. 8d). Patients scheduled for a screening colonoscopy were stratified as high vs. low cruciferous vegetable consumers based on a validated questionnaire [28]. In PBMCs obtained immediately prior to colonoscopy, *p16* mRNA levels were significantly higher for subjects reporting >5 vs. 0–1 servings of cruciferous vegetables per week (Fig. 8e). We next examined the corresponding colon biopsies for selected proteins of interest, i.e.*,* p16, HDACs, and histone acetylation (Fig. 8f). With higher cruciferous vegetable intake, histone acetylation and p16 expression were increased, whereas HDAC1 levels were unchanged. Based on densitometry measurements of immunoblots, a significant inverse association was observed for p16 and HDAC3 in colon biopsies (Fig. 8g), whereas p16 and acetyl histone H4 lysine 12 (AcH4K12) (Fig. 8h) and HDAC3 and Nrf2 (Fig. 8i) were positively correlated.

## Discussion

SFN was first identified as a potent inducer of phase 2 enzymes, acting via the Nrf2 pathway to induce detoxification pathways that favor carcinogen excretion and elimination from the body [14, 15]. However, SFN also can be effective in post-initiation protocols [29], independent of carcinogen exposure. This is exemplified by the tumor suppression observed for SFN in genetic models, such as the adenomatous polyposis coli/multiple intestinal neoplasia (Apc^Min/+^) mouse, in which HDAC inhibition was identified as a contributing mechanism [10]. Therefore, we examined the interplay between two key mechanisms implicated in the SFN antitumor activity, namely, Nrf2 induction and HDAC inhibition. Although pharmacological HDAC inhibitors have been tested in preclinical models of colon cancer [30], the role of Nrf2 was not examined. We specifically sought to test the hypothesis that Nrf2 status might affect HDAC3 protein expression levels in colon tumors, and thus the inhibitory response to SFN acting preferentially on HDAC3 [20, 21].

As in the Apc^Min/+^ mouse [10, 31], SFN suppressed tumorigenesis in the DMH model, and this was accompanied by reduced HDAC activity and HDAC3 protein expression in the colon tumors. These findings are in accordance with data from human colon cancer cells showing HDAC inhibition by SFN and its metabolites [11, 20], and by other dietary isothiocyanates [21], to involve HDAC3 protein turnover. Metabolites implicated in the HDAC3 turnover mechanism [11, 20, 21], such as SFN-Cys and SFN-NAC, were detected in tissues of SFN-treated mice, as reported [22]. The corroboration of HDAC3 as a target of SFN in vivo is important considering the critical role of this HDAC in regulating colon cancer growth and tumorigenesis [3]. Loss of tumor suppression in mice fed with SFN on alternating days (Fig. 1) might be related to the inability to sustain high enough SFN metabolite levels for effective HDAC3 inhibition. Indeed, SFN metabolites are cleared within 24 h in mice [22], as in human subjects consuming BSE (Fig. 8b).

Nrf2-deficient mice are generally more sensitive to carcinogens and agents that trigger chronic inflammation [32–35]. However, Nrf2^−/+^ mice treated with DMH and observed for up to 35 weeks had a significantly reduced tumor burden and lower HDAC protein expression compared with WT animals. The differential response to the carcinogen likely was not attributable to genes that regulate DMH metabolism (*Cyp2E1*) or DNA repair (*Mgmt*), since their expression was similar in WT and Nrf2^−/+^ mouse colon (Additional file 2: Figure S4). In a recent study using urethane to initiate lung tumors [16], resistance to tumor growth was observed in Nrf2-deficient mice compared with WT. The enhanced tumor growth in WT mice adds to the discussion on pros vs. cons of Nrf2 signaling in different stages of cancer development [17, 36].

From the gene expression arrays, qRT-PCR and immunoblotting experiments, tumor suppressors p21 and p15 were induced only marginally, if at all, by SFN in the preclinical model reported here, in marked contrast to p16. For example, in gene expression arrays (Additional file 1: Table S1), p21 and p15 were attenuated slightly in the colon tumors from SFN-treated mice, whereas p16 was induced greater than 20-fold. Although p16 more typically is considered a tumor suppressor protein, high *Cdkn2a/p16* levels have been detected in mouse colon tumors induced by azoxymethane, a metabolite of DMH [37], and in human benign tumors and high-grade malignancies [38]. Overexpression of p16 has been linked to the so-called oncogene-induced senescence (OIS) in benign tumors or as a mechanism to arrest uncontrolled proliferation in advanced cancers [38]. Early upregulation of p16 in some tumors might represent an attempt to correct for one or more dysregulated signaling pathways. We focused on *p16* as a major target dysregulated in DMH-induced colon tumors and noted that SFN increased *p16* expression in tumors of WT mice but had the opposite effect in Nrf2^−/+^ mice. Based on evidence that HDAC inhibition activates *p16* [39–41], we confirmed HDAC3 interactions on *p16* to be higher in colon tumors of WT vs. Nrf2^−/+^ mice (Fig. 6f), and inversely associated with *p16* mRNA levels (Fig. 6a). In human colon cancer cells, HDAC3 knockdown increased *p16* levels to a similar extent as SFN treatment, whereas Keap1 knockdown had no effect on *p16* (Fig. 7)*.* This suggested that *p16* is regulated by HDAC3 but is not a direct target of Nrf2.

What, then, connects Nrf2 genetic background to altered HDAC3 levels on *p16*? A working model can be proposed (Additional file 2: Figure S5). We speculate that Nrf2 deficiency in mice, through mechanisms that remain to be clarified, attenuates the overall rate of colon tumor growth and global HDAC levels within the tumor. This in turn diminishes HDAC interactions on key genes dysregulated during tumor development, regardless of whether or not they are directly regulated by Nrf2 binding. Mechanisms affecting promoter methylation and transcription factor access [42–45] might influence which genes are most altered, and the ultimate response to SFN treatment.

**Fig. 9:**
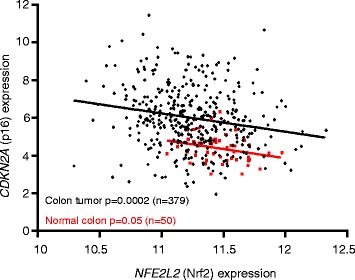
Nrf2 and p16 are inversely associated in human colon tumors and normal colon. The Cancer Genome Atlas (TCGA, https://tcga-data.nci.nih.gov/tcga/) was mined for *NFE2L2* (*Nrf2*) and *CDKN2A* (*p16*) mRNA expression data in colon tumors (*black*) and normal colon (*red*)

The BSE supplement is being evaluated in several human trials (clinicaltrials.gov). In the current investigation, SFN metabolites were detected in plasma shortly after BSE intake, and in circulating PBMCs, there was increased p16 and transiently decreased HDAC3 expression. As predicted from the preclinical model, in human colon biopsies HDAC3 was inversely associated with p16 (Fig. 8g), and positively correlated with Nrf2 (Fig. 8i). We were interested in the variability of Nrf2 expression in normal colon biopsies (Fig. 8f). In The Cancer Genome Atlas (TCGA) database [46], *NFE2L2* (*Nrf2*) levels also varied markedly in normal colon and in colon tumors and were inversely correlated with *Cdkn2a/p16* (Fig. 9), in accordance with data from cell-based, preclinical, and translational studies reported here. We are now recruiting additional human volunteers to obtain both polyps and adjacent normal colon biopsies, seeking to corroborate the proposed cross-talk between Nrf2, HDAC3, and p16.

## Conclusions

Nrf2 status in the mouse colon appears to serve as an arbiter of overall colon tumor growth, HDAC protein expression in colon tumors, and the response to HDAC inhibitor treatment mediated by downstream molecular targets such as p16. A key issue will be the extent to which Nrf2 status influences HDAC levels and p16 expression at different stages of human colon cancer development and the ultimate response to pan-HDAC or HDAC-selective inhibitors. This could have implications beyond the treatment of colorectal cancer, for example, in other solid tumors and hematological malignancies currently undergoing clinical evaluation with HDAC inhibitors [47]. In the broad context of these various clinical trials and their overall aims, we believe that Nrf2 status is worthy of further investigation as a possible determinant of tumor growth and HDAC inhibitor responsiveness.

## Methods

### Animals and diets

Male WT or Nrf2^−/+^ mice at 8–10 weeks of age were randomized to 25 mice/group (pilot study) or 35 mice/group (main study). Tumors were induced by 1,2-dimethylhydrazine (DMH, Sigma-Aldrich), as reported [19]. After DMH treatment, mice continued on regular AIN93 diet for an additional week before administering AIN93 diet or AIN93 diet supplemented with 400 ppm d,l-SFN (Toronto Research Chemicals, Inc.), either continuously or on alternating days. At end of the study, the colon was removed, and tumors were scored for number, size, and position by individuals blinded to the treatment. Tumor volume was calculated using the formula *xy*^2^ * 0.5 (*x* = long diameter, *y* = short diameter). Total tumor burden per animal was calculated by adding the individual tumor volumes. Tumor and adjacent normal-looking tissue was removed and one portion was fixed in 10 % buffered formalin, while the other portion was flash-frozen in liquid nitrogen and stored at −80 °C. The work was approved by the Institutional Animal Care and Use Committee.

### HDAC activity

HDAC activity was measured using the FLUOR DE LYS assay, as reported [10, 20].

### Immunoblotting

Frozen samples of colon tumors and adjacent tissue were thawed and subjected to immunoblotting using the methodology described previously [20, 21, 48]. Antibodies were for HDAC1 and HDAC3 (Santa Cruz), p16 (Proteintech), acetyl histone H4K12 and histone H4 (Cell Signaling), and β-actin (Sigma-Aldrich).

### PCR arrays and qPCR

RT^2^ Profiler arrays were run as per manufacturer’s instructions (SA Biosciences, Qiagen, Valencia, CA, USA) on colon tumor samples (pooled, *n* = 6) and matched controls (pooled, *n* = 6). From the threshold cycle (*C*_t_) value, the relative gene expression of each target was normalized to human β-actin gene (*ACTB*) or murine β-actin gene (*Actb*) (β-actin gene in human and mouse, respectively). Two or more separate qRT-PCR experiments were performed to validate targets of interest*,* as reported [48].

### ChIP assays

The ChIP-IT Express Enzymatic kit (Active Motif, Carlsbad, CA) was used, as reported [49]. Frozen tissue (100 mg) was cut into pieces, cross-linked with formaldehyde, and homogenized in order to isolate the nuclear fraction. DNA fragmentation was performed via enzymatic shearing, using a proprietary cocktail (Active Motif) that randomly cleaves between nucleosomes, generating DNA fragments of ~500 bp. Ten microliters of fragmented chromatin was kept as input while the remaining was immunoprecipitated (IP) with anti-HDAC3 antibody (Santa Cruz). After reversing the cross-linking and proteinase treatment, DNA was purified using QIAquick PCR Purification kits (Qiagen). PCR was run on a Roche LightCycler 480 II with preincubation for 5 min at 95 °C, then 45 cycles at 95 °C for 10 s, 60 °C for 10 s, and 72 °C for 10 s. Each experiment was repeated at least twice.

### Immunohistochemistry

Formalin-fixed paraffin-embedded mouse colon tumor and adjacent normal tissue were processed for immunohistochemistry as reported [48]. Slides containing 5 μm sections were rehydrated and placed in an Autostainer (Dako). After primary antibody to p16 (Proteintech) for 30 min and One-Step HRP Polymer anti-IgG (ImmunoBioscience) for 7 min, Nova Red (Vector Labs) was applied for 5 min followed by hematoxylin (Dako). Images were acquired on a Nikon E400 microscope equipped with a CCD camera.

### Knockdown experiments

HCT116 cells were obtained from American Type Culture Collection (Manassas, VA, USA) and validated as reported [50]. Cells were transfected for 48 h with HDAC3 siRNA (Trilencer-27, OriGene), Keap1 siRNA (Sigma-Aldrich), control siRNA (OriGene), or Lipofectamine 2000 alone, using the manufacturer’s protocol (Invitrogen).

### Human studies

#### Phase “0” trial

A pilot intervention study was performed based on a prior protocol [12]. Ten healthy subjects avoided cruciferous vegetables, starting 1 week before day 1 of the study and continuing through day 14. After an overnight fast, volunteers ate a standardized breakfast together with a broccoli sprout extract (BSE) supplement (IND #111736, 200 μmol SFN equivalents, *n* = 5) or a placebo (*n* = 5), once a day for 7 days. BSE and placebo were obtained from Johns Hopkins University (Baltimore, MD, USA), with SFN content validated by LC-MS/MS, as reported [27]. The Institutional Review Board (IRB) approved the protocol, and all participants provided written consent.

Whole blood was collected into ethylene diamine tetraacetic acid (EDTA) Vacutainers (VWR, Radnor, PA, USA) at 0, 1, 3, and 6 h post-consumption on days 1 and 7 and on days 8, 9, and 14. After centrifuging at 2000 rpm for 30 min, plasma was removed, acidified with trifluoroacetic acid, and stored at −80 °C. Samples were analyzed for SFN metabolites as previously described [27]. The remaining whole blood was processed to isolate peripheral blood mononuclear cells (PBMC), as described previously [12], and frozen at −80 °C.

#### Screening colonoscopy study

Men and women aged >50 years and scheduled for a screening colonoscopy were recruited based on cruciferous vegetable consumption (*n* = 28). Recruitment and data collection were implemented through the Oregon Clinical and Translational Research Institute and the Oregon Health & Science University Cancer Institute, with IRB approval and written consent from each participant. Subjects completed a validated [28] cruciferous vegetable food frequency questionnaire (CVFFQ) and had three 24-h dietary recalls over a 3-week period. The CVFFQ assessed intake over the previous 12 months, relating to number of servings, serving size, intake of raw and cooked vegetables, method of cooking, and use of condiments. Data from the CVFFQ were analyzed by the Arizona Diet, Behavioral, and Quality of Life Assessment Center, University of Arizona, Tucson, AZ, USA. Volunteers were stratified into low (0–1 serving/week, *n* = 5) and high (≥5 servings/week, *n* = 23) consumers. Dietary recalls were used to provide information on possible changes in diet between the CVFFQ and the clinic visit. Blood was obtained and processed as in the BSE trial. In addition, two biopsies of rectal colon and two of proximal colon were taken with standard forceps and placed in formalin or flash-frozen in liquid nitrogen.

### Statistics

Results were expressed as mean ± SD. Analysis of variance (ANOVA) was used for group comparisons, followed by Bonferroni’s multiple comparison test (GraphPad Prism v 5.04). Student’s *t* test was used for paired comparisons, with *P* < 0.05 considered as significant.

## Additional files

Additional file 1: Table S1. Gene expression changes in mouse colon. The Table shows gene expression data of '84 genes' in the RT2 Profiler array as described in Methods. Each colum (as labeled) provides a list of the different genes, gene names, and the gene expression data (relative to ACTB) normalized to normal colon in WT vehicle control mice calculated using the RT2 Profier PCR Array Data Analysis software. Each row provides data for a single gene, positive values indicating higher expression whereas negative values indicating lower gene expression compared to normal colon in WT vehicle controls. (DOCX 30 kb) Additional file 2: Figure S1. Validation of gene array data in mouse colon tumors and normal colon. **a**–**d** Relative mRNA expression of selected genes normalized to the β-actin gene (*Actb*) in normal colonic mucosa, and **e**–**h** in DMH-induced colon tumors. Data = mean ± SD (*n* = 3). Red arrow, *p16*. **Figure S2.** HDAC3 interactions with *Esr1* are unaffected by SFN. Primers were designed to interrogate the promoter region adjacent to the transcriptional start site of *Esr1*, a gene strongly overexpressed in DMH-induced colon tumors (see Fig. 5b). ChIP data = mean ± SD from three independent ChIP assays. *Esr1* served as a negative control for the ChIP assays with *p16* (see Fig. 6f). **Figure S3.** SFN altered HDAC3 and p16 expression in mouse splenocytes. Mice (*n* = 3) were administered a single oral gavage of 200 μmol SFN, and mononuclear cells were isolated from mouse spleen at 6 h, as reported earlier [12]. **a** Whole cell lysates were immunoblotted for HDAC3, p16, and AcH4K12 with densitometry data normalized to β-actin. **b** A colon tumor from DMH-treated mice was included as a reference control. **Figure S4.** No change in the expression of genes involved in DMH activation (*Cyp2E1*) and DNA repair (*Mgmt*). Relative mRNA expression levels were normalized to the β-actin gene, *Actb*. Data = mean ± SD (*n* = 3). **Figure S5.** Working model for Nrf2 status impacting tumor growth, HDAC3 levels, and p16 induction. (PPTX 276 kb) Additional file 3: Table S2. SFN and its metabolites in human plasma following ingestion of a BSE supplement. The levels of "SFN and its metabolites" in human plasma was measured using Liquid Chromatrography-Mass Spectrometry (LC-MS) methods, as described previously [27]. Calculated pharmacokinetic parameters - Tmax, Cmax, AUC, and  half-life (mean) values are shown in 'rows' for each SFN metabolite, measured on Days 1 and 7, shown in respective columns. (DOCX 19 kb)

## Abbreviations

AcH4K12: acetyl histone H4 lysine 12
*ACTB*: human β-actin gene
*Actb*: murine β-actin gene
Apc^Min/+^: adenomatous polyposis coli/multiple intestinal neoplasia
BSE: broccoli sprout extract
Cdkn1a/p21: cyclin-dependant kinase inhibitor 1A
*Cdkn2a/p16*: cyclin-dependant kinase inhibitor 2A
Cdkn2b/p15: cyclin-dependent kinase inhibitor 2B
ChIP: chromatin immunoprecipitation
CVFFQ: cruciferous vegetable food frequency questionnaire
Cyp2E1: cytochrome P450 isoform 2E1
DMH: 1,2-dimethylhydrazine
EDTA: ethylene diamine tetraacetic acid
*Esr1*: estrogen receptor 1
*Ets1*: E26 avian leukemia oncogene 1,5′ domain
HDAC: histone deacetylase
HDAC1: histone deacetylase 1
HDAC3: histone deacetylase 3
*HO1*: heme oxygenase-1
Igf2r: insulin-like growth factor 2 receptor
Keap1: Kelch-like ECH-associated protein 1
Met: Met proto-oncogene
MGMT: O^6^-alkylguanine DNA methyltransferase
Mlh1: mutL homolog 1
Nrf2: nuclear factor erythroid 2 (NF-E2)-related factor 2
OIS: oncogene-induced senescence
Tp53: transformation-related protein 53
PBMCs: peripheral blood mononuclear cells
ppm: parts per million
Prkca: protein kinase C, alpha
Runx1: runt-related transcription factor 1
Serpin b5: serine peptidase inhibitor
SFN: sulforaphane
SFN-CG: SFN-cysteine-glycine
SFN-Cys: SFN-cysteine
SFN-GSH: SFN-glutathione
SFN-NAC: SFN-*n*-acetylcysteine
TCGA: The Cancer Genome Atlas
WT: wild type
