# Supplementary material for: Nrf2 status affects tumor growth, HDAC3 gene promoter associations, and the response to sulforaphane in the colon
Source: Clin Epigenetics. 2015 Sep 18;7(1):102. doi: 10.1186/s13148-015-0132-y (PMC4575421; doi:10.1186/s13148-015-0132-y)
Supplement: Additional file 1: Table S1. — Gene expression changes in mouse colon. The Table shows gene expression data of '84 genes' in the RT2 Profiler array as described in Methods. Each colum (as labeled) provides a list of the different genes, gene names, and the gene expression data (relative to ACTB) normalized to normal colon in WT vehicle control mice calculated using the RT2 Profier PCR Array Data Analysis software. Each row provides data for a single gene, positive values indicating higher expression whereas negative values indicating lower gene expression compared to normal colon in WT vehicle controls. (DOCX 30 kb) [file 13148_2015_132_MOESM1_ESM.docx]

Additional file 1: Table S1

| **No.** | **Gene Symbol** | **Gene Name** | **Fold change compared to normal colon in**  **WT vehicle controls** | | | | | | | |
| --- | --- | --- | --- | --- | --- | --- | --- | --- | --- | --- |
|  |  |  | **WT mice** | | | | **Nrf2^-/-^ mice** | | | |
|  |  |  | **Normal** | | **Tumor** | | **Normal** | | **Tumor** | |
|  |  |  | -SFN | +SFN | -SFN | +SFN | -SFN | +SFN | -SFN | +SFN |
| 1 | Abl1 | C-abl oncogene 1, non-receptor tyrosine kinase | 1.00 | 1.57 | 3.41 | -1.24 | 1.38 | 1.33 | 7.52 | 1.3 |
| 2 | Akt1 | Thymoma viral proto-oncogene 1 | 1.00 | 1.21 | -1.01 | 1.06 | -1.09 | -1.21 | 3.1 | -1.19 |
| 3 | Apc | Adenomatosis polyposis coli | 1.00 | 1.27 | -1.1 | -1.2 | -1.73 | -3.76 | -2.3 | -2.95 |
| 4 | Atm | Ataxia telangiectasia mutated homolog (human) | 1.00 | 1.53 | 1.52 | 1.34 | 1.27 | 1.66 | 1.34 | 1.67 |
| 5 | Bax | Bcl2-associated X protein | 1.00 | 1.65 | 2.04 | 1.83 | 1.25 | 1.22 | 9.99 | 1.69 |
| 6 | Bcl2 | B-cell leukemia/lymphoma 2 | 1.00 | 2.16 | 1.21 | -1.04 | 1.11 | -2.16 | 1.48 | 1.24 |
| 7 | Bcl2l1 | Bcl2-like 1 | 1.00 | 1.32 | 1.32 | 1.6 | -1.85 | -1.77 | 3.18 | 1.38 |
| 8 | Bcr | Breakpoint cluster region | 1.00 | 2.31 | 1.05 | 1.03 | -2.17 | -1.45 | -1.58 | -1.27 |
| 9 | Brca1 | Breast cancer 1 | 1.00 | -1.8 | 2.08 | 2.5 | 1.3 | -1.65 | 3.03 | 1.96 |
| 10 | Brca2 | Breast cancer 2 | 1.00 | 3.14 | 2.41 | 2.23 | 2.08 | 1.71 | 7.46 | 2.28 |
| 11 | Casp8 | Caspase 8 | 1.00 | 3.23 | 2.07 | 2.55 | 2.31 | 2.13 | 1.29 | 2.31 |
| 12 | Ccnd1 | Cyclin D1 | 1.00 | 1.99 | 2.68 | 3.16 | 2.6 | 1.77 | 1.91 | 2.00 |
| 13 | Cdh1 | Cadherin 1 | 1.00 | -1.04 | -1.1 | -1.43 | -1.45 | -1.64 | 1.69 | -1.57 |
| 14 | Cdk4 | Cyclin-dependent kinase 4 | 1.00 | -1.09 | 3.97 | 3.05 | 1.30 | -1.13 | 4.00 | 3.71 |
| 15 | Cdkn1a | Cyclin-dependent kinase inhibitor 1A (p21) | 1.00 | 1.08 | -1.6 | -1.48 | -2.1 | -1.49 | 2.13 | -2.17 |
| 16 | Cdkn2a | Cyclin-dependent kinase inhibitor 2A | 1.00 | -1.22 | 12.8 | 20.53 | 2.69 | 1.6 | 19.9 | 8.46 |
| 17 | Cdkn2b | Cyclin-dependent kinase inhibitor 2B (p15, inhibits CDK4) | 1.00 | 2.14 | -5.46 | -2.1 | 1.67 | 1.42 | -3.63 | -3.39 |
| 18 | Cdkn3 | Cyclin-dependent kinase inhibitor 3 | 1.00 | 1.67 | 1.1 | -1.08 | 1.4 | 1.65 | 1.4 | 1.75 |
| 19 | Ctnnb1 | Catenin (cadherin associated protein), beta 1 | 1.00 | 1.2 | -1.02 | 1.07 | -1.68 | -1.48 | -1.34 | -1.27 |
| 20 | E2f1 | E2F transcription factor 1 | 1.00 | 1.66 | 1.49 | 1.58 | 1.99 | -1.26 | 1.19 | 1.28 |
| 21 | Egf | Epidermal growth factor | 1.00 | 1.48 | -3.16 | -3.51 | 1.11 | 1.67 | 2.41 | -4.47 |
| 22 | Elk1 | ELK1, member of ETS oncogene family | 1.00 | 2.69 | 1.37 | 1.45 | 1.16 | 1.03 | 1.56 | 1.37 |
| 23 | Erbb2 | V-erb-b2 erythroblastic leukemia viral oncogene homolog 2 | 1.00 | 1.46 | -2.22 | -1.51 | -1.83 | -1.25 | -1.38 | -2.1 |
| 24 | Esr1 | Estrogen receptor 1 (alpha) | 1.00 | 1.91 | 11.24 | 7.01 | -1.16 | -1.75 | 17.75 | 7.26 |
| 25 | Ets1 | E26 avian leukemia oncogene 1, 5' domain | 1.00 | 2.08 | 2.64 | 1.53 | 1.72 | -1.45 | 9.99 | 2.03 |
| 26 | Fhit | Fragile histidine triad gene | 1.00 | -1.09 | 2.13 | 1.85 | -1.19 | 1.25 | 1.91 | 1.79 |
| 27 | Fos | FBJ osteosarcoma oncogene | 1.00 | -1.42 | -2.45 | -2.14 | -3.94 | -4.56 | -2.23 | -2.57 |
| 28 | Foxd3 | Forkhead box D3 | 1.00 | -1.16 | -1.14 | 1.04 | -1.57 | -1.48 | -1.91 | 1.27 |
| 29 | Hgf | Hepatocyte growth factor | 1.00 | -1.39 | 2.68 | 2.55 | 1.95 | -1.13 | 2.68 | 3.14 |
| 30 | Hic1 | Hypermethylated in cancer 1 | 1.00 | 1.56 | 1.77 | 1.62 | 1.26 | -1.32 | 4.44 | 1.66 |
| 31 | Hras1 | Harvey rat sarcoma virus oncogene 1 | 1.00 | 7.52 | 1.66 | 1.39 | 1.93 | 1.21 | 3.2 | 2.03 |
| 32 | Igf2r | Insulin-like growth factor 2 receptor | 1.00 | 1.4 | -1.97 | -1.34 | -1.47 | -1.45 | 1.56 | -1.97 |
| 33 | Jak2 | Janus kinase 2 | 1.00 | 1.25 | 2.93 | 2.3 | 1.65 | -1.1 | 4.53 | 2.71 |
| 34 | Jun | Jun oncogene | 1.00 | 1.59 | 1.46 | 1.39 | -1.39 | -1.6 | 5.62 | 2.01 |
| 35 | Junb | Jun-B oncogene | 1.00 | -1.51 | -2.33 | -1.96 | -2.53 | -2.19 | 1.66 | -3.18 |
| 36 | Jund | Jun proto-oncogene related gene d | 1.00 | -1.58 | -1.84 | -1.07 | -2.85 | -2.36 | -1.54 | -1.75 |
| 37 | Kit | Kit oncogene | 1.00 | -1.36 | 1.78 | 1.69 | 1.53 | -1.12 | 3.23 | 2.3 |
| 38 | Kitl | Kit ligand | 1.00 | 1.95 | -1.03 | -2.51 | -2.5 | -1.97 | 2.79 | -1.75 |
| 39 | Kras | V-Ki-ras2 Kirsten rat sarcoma viral oncogene homolog | 1.00 | 1.35 | -2.95 | -4.35 | 1.29 | -1.07 | -1.4 | -2.39 |
| 40 | Mcl1 | Myeloid cell leukemia sequence 1 | 1.00 | 1.01 | 1.35 | 1.55 | 1.12 | -1.01 | 1.93 | 2.64 |
| 41 | Mdm2 | Transformed mouse 3T3 cell double minute 2 | 1.00 | -1.2 | -1.66 | -1.88 | -1.16 | 1.02 | 1.59 | -1.66 |
| 42 | Men1 | Multiple endocrine neoplasia 1 | 1.00 | -1.15 | -2.01 | -1.92 | -1.32 | 1.06 | 1.22 | -1.93 |
| 43 | Met | Met proto-oncogene | 1.00 | 1.53 | 1.39 | 1.44 | -1.12 | -1.2 | 3.43 | 1.33 |
| 44 | Mgmt | O-6-methylguanine-DNA methyltransferase | 1.00 | 1.55 | 1.15 | 1.16 | -1.96 | -2.16 | -1.46 | 1.16 |
| 45 | Mlh1 | MutL homolog 1 (E. coli) | 1.00 | -1.93 | -5.03 | -2.2 | -3.36 | -4.69 | -13.74 | -3.53 |
| 46 | Mos | Moloney sarcoma oncogene | 1.00 | 1.36 | 1.24 | 1.21 | 1.85 | -1.02 | 2.35 | 1.34 |
| 47 | Myb | Myeloblastosis oncogene | 1.00 | -1.41 | 1.07 | 1.48 | 2.83 | 1.21 | -4.41 | 1.42 |
| 48 | Myc | Myelocytomatosis oncogene | 1.00 | 2.46 | -4.82 | -2.2 | 5.7 | 3.39 | 12.04 | 1.42 |
| 49 | Mycn | V-myc myelocytomatosis viral related oncogene | 1.00 | -1.58 | 1.06 | 1.37 | 1.1 | -1.07 | -2.17 | 1.09 |
| 50 | Nf1 | Neurofibromatosis 1 | 1.00 | -2.23 | 2.51 | 3.34 | -1.13 | -2.51 | 1.39 | 3.46 |
| 51 | Nf2 | Neurofibromatosis 2 | 1.00 | -1.13 | -1.41 | 2.03 | 2.6 | 1.92 | 1.31 | 1.13 |
| 52 | Nfkb1 | Nuclear factor of kappa light polypeptide gene enhancer in B-cells 1, p105 | 1.00 | 1.05 | 1.06 | 1.17 | 1.33 | 1.31 | 3.41 | -1.06 |
| 53 | Nfkbia | Nfkb1 inhibitor, alpha | 1.00 | -1.05 | 1.01 | -1.29 | -1.01 | -1.03 | 1.26 | -1.12 |
| 54 | Nras | Neuroblastoma ras oncogene | 1.00 | 1.26 | -1.13 | 1.01 | 1.06 | -1.28 | -1.72 | 1.06 |
| 55 | Pik3c2a | Phosphatidylinositol 3-kinase, C2 domain containing, alpha polypeptide | 1.00 | 1.52 | -1.56 | -1.79 | -1.05 | 1 | -2.16 | -1.75 |
| 56 | Pik3ca | Phosphatidylinositol 3-kinase, catalytic, alpha polypeptide | 1.00 | 1.47 | 1.55 | 1.39 | -1.03 | -1.44 | 1 | 1.29 |
| 57 | Pml | Promyelocytic leukemia | 1.00 | -1.54 | -1.75 | -1.2 | -2.41 | -1.68 | -2.6 | -1.69 |
| 58 | Prkca | Protein kinase C, alpha | 1.00 | -1.29 | -1.67 | -1.26 | -2.2 | -1.79 | -1.16 | -1.85 |
| 59 | Raf1 | V-raf-leukemia viral oncogene 1 | 1.00 | -2.39 | -1.97 | -1.24 | -2.53 | -4.99 | -2.08 | -1.77 |
| 60 | Rara | Retinoic acid receptor, alpha | 1.00 | -1.8 | -13.55 | -5.5 | -1.85 | -1.6 | -6.36 | -11.08 |
| 61 | Rassf1 | Ras association (RalGDS/AF-6) domain family member 1 | 1.00 | -1.84 | -1.31 | -1.27 | -1.96 | -1.67 | -1.05 | -1.68 |
| 62 | Rb1 | Retinoblastoma 1 | 1.00 | -1.36 | -1.44 | 1.71 | -1.59 | -2.66 | 1.53 | -1.25 |
| 63 | Rel | Reticuloendotheliosis oncogene | 1.00 | -1.13 | -1.01 | -1.21 | 1.31 | -1.17 | 1.14 | 1.09 |
| 64 | Ret | Ret proto-oncogene | 1.00 | -1.2 | -1.72 | -1.97 | -1.36 | -1.21 | -3.14 | -1.46 |
| 65 | Ros1 | Ros1 proto-oncogene | 1.00 | -1.32 | -1.32 | -1.21 | -2.39 | -2.66 | -2.85 | -1.48 |
| 66 | Runx1 | Runt related transcription factor 1 | 1.00 | 1.01 | -3.41 | -1.93 | 1.01 | 1.04 | 1.83 | -3.66 |
| 67 | Runx3 | Runt related transcription factor 3 | 1.00 | -1.04 | 2.35 | 2.11 | -1.89 | -1.67 | 3.2 | 1.32 |
| 68 | S100a4 | S100 calcium binding protein A4 | 1.00 | 1.51 | -1.05 | -1.17 | 1.84 | -1.91 | 4.38 | -1.08 |
| 69 | Serpinb5 | Serine (or cysteine) peptidase inhibitor, clade B, member 5 | 1.00 | 1.69 | -2.62 | -2.43 | 2.83 | 3.16 | -4.76 | -1.93 |
| 70 | Sh3pxd2a | SH3 and PX domains 2A | 1.00 | -4.53 | -8.88 | -2.16 | -2.14 | -2.81 | -40.79 | -8.4 |
| 71 | Smad4 | MAD homolog 4 (Drosophila) | 1.00 | -2.23 | -2.13 | -1.54 | -1.25 | -1.72 | 1.05 | -2.77 |
| 72 | Src | Rous sarcoma oncogene | 1.00 | -1.55 | -1.57 | -1.1 | 1.06 | 1.01 | -5.86 | -1.92 |
| 73 | Stat3 | Signal transducer and activator of transcription 3 | 1.00 | -1.37 | 1.01 | 1.33 | -1.47 | -1.1 | 1.57 | -1.61 |
| 74 | Stk11 | Serine/threonine kinase 11 | 1.00 | -1.26 | 1.54 | 1.57 | -1.08 | -1.27 | 4.53 | 1.47 |
| 75 | Tgfb1 | Transforming growth factor, beta 1 | 1.00 | -1.02 | -1.12 | 1.26 | 1.6 | 1.26 | 3.39 | 1.67 |
| 76 | Tnf | Tumor necrosis factor | 1.00 | 1.65 | 2.43 | 1.87 | 1.93 | -1.18 | 8.28 | 2.17 |
| 77 | Trp53 | Transformation related protein 53 | 1.00 | -2.55 | 1.27 | -1.09 | -1.21 | -2.06 | 1.84 | 2.22 |
| 78 | Trp73 | Transformation related protein 73 | 1.00 | -1 | 2.51 | 2.33 | 1.2 | 1.09 | 8.69 | 2.45 |
| 79 | Tsc1 | Tuberous sclerosis 1 | 1.00 | -1.13 | -2.79 | -2 | -1.85 | -1.66 | 1.32 | -2.41 |
| 80 | Vhl | Von Hippel-Lindau tumor suppressor | 1.00 | -1.27 | 1.01 | -1.19 | 1.51 | 1.26 | -2.14 | 1.25 |
| 81 | Wt1 | Wilms tumor 1 homolog | 1.00 | -1.72 | -4.44 | -3.63 | 6.87 | 4.03 | 14.52 | -6.28 |
| 82 | Wwox | WW domain-containing oxidoreductase | 1.00 | -1.59 | 1.96 | 1.16 | -2 | -1.13 | -1.13 | 2.19 |
| 83 | Xrcc1 | X-ray repair complementing defective repair in Chinese hamster cells 1 | 1.00 | -1.58 | 1.28 | 1.89 | 1.35 | -1.04 | -1.02 | 1.24 |
| 84 | Zhx2 | Zinc fingers and homeoboxes 2 | 1.00 | -1.78 | 1.16 | 1.16 | 1.13 | -1.04 | 2.53 | -1.16 |
