# Supplementary material for: Nrf2 status affects tumor growth, HDAC3 gene promoter associations, and the response to sulforaphane in the colon
Source: Clin Epigenetics. 2015 Sep 18;7(1):102. doi: 10.1186/s13148-015-0132-y (PMC4575421; doi:10.1186/s13148-015-0132-y)
Supplement: Additional file 2: Figure S1. — Validation of gene array data in mouse colon tumors and normal colon. a–d Relative mRNA expression of selected genes normalized to the β-actin gene (Actb) in normal colonic mucosa, and e–h in DMH-induced colon tumors. Data = mean ± SD (n = 3). Red arrow, p16. Figure S2. HDAC3 interactions with Esr1 are unaffected by SFN. Primers were designed to interrogate the promoter region adjacent to the transcriptional start site of Esr1, a gene strongly overexpressed in DMH-induced colon tumors (see Fig. 5b). ChIP data = mean ± SD from three independent ChIP assays. Esr1 served as a negative control for the ChIP assays with p16 (see Fig. 6f). Figure S3. SFN altered HDAC3 and p16 expression in mouse splenocytes. Mice (n = 3) were administered a single oral gavage of 200 μmol SFN, and mononuclear cells were isolated from mouse spleen at 6 h, as reported earlier [12]. a Whole cell lysates were immunoblotted for HDAC3, p16, and AcH4K12 with densitometry data normalized to β-actin. b A colon tumor from DMH-treated mice was included as a reference control. Figure S4. No change in the expression of genes involved in DMH activation (Cyp2E1) and DNA repair (Mgmt). Relative mRNA expression levels were normalized to the β-actin gene, Actb. Data = mean ± SD (n = 3). Figure S5. Working model for Nrf2 status impacting tumor growth, HDAC3 levels, and p16 induction. (PPTX 276 kb) [file 13148_2015_132_MOESM2_ESM.pptx]

## Slide 1
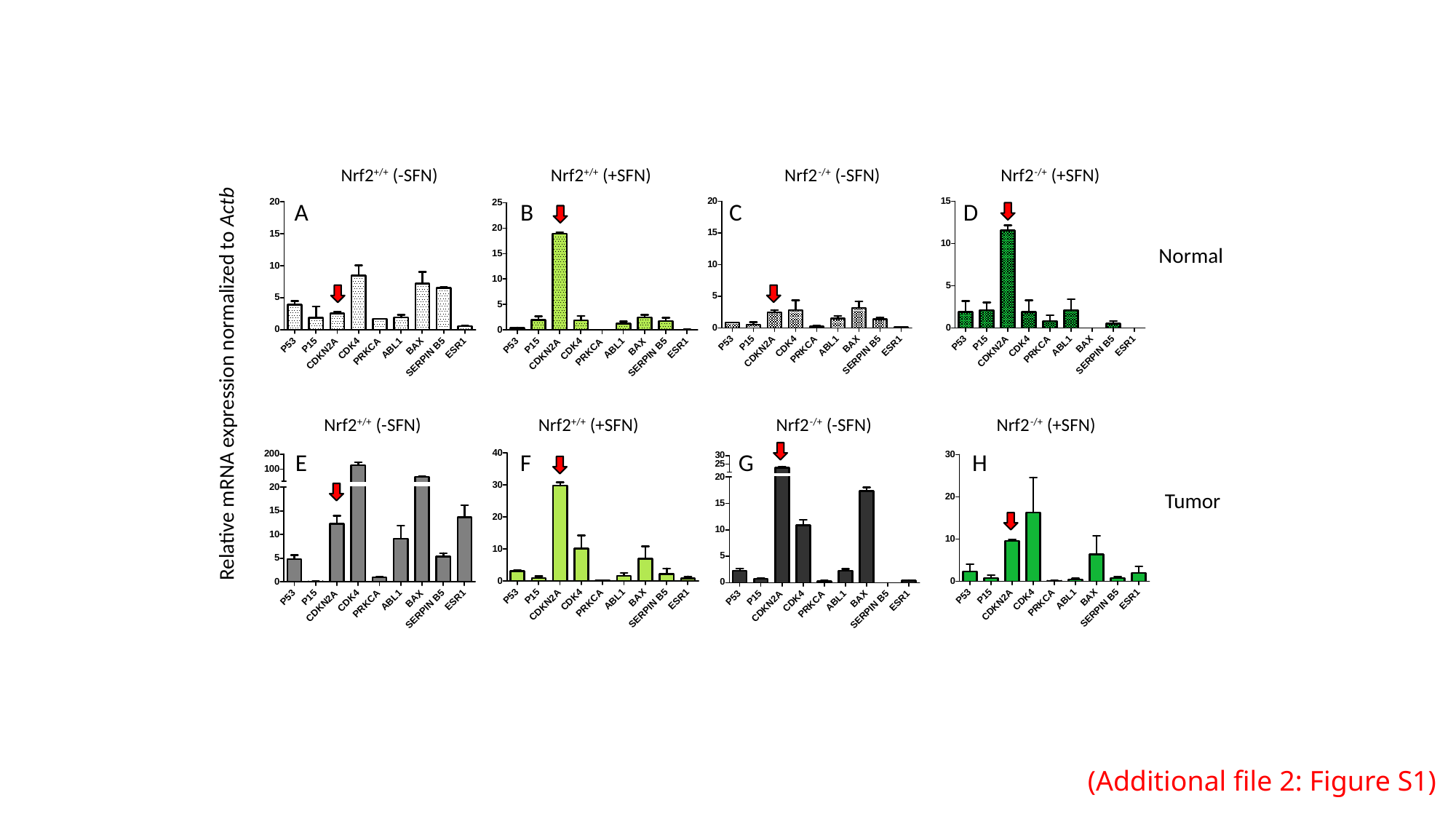

Nrf2+/+ (-SFN) 	 Nrf2+/+ (+SFN) Nrf2-/+ (-SFN) Nrf2-/+ (+SFN)
A
B
C
D
Normal
Relative mRNA expression normalized to Actb
 Nrf2+/+ (-SFN) 		Nrf2+/+ (+SFN) Nrf2-/+ (-SFN) Nrf2-/+ (+SFN)
E
F
G
H
Tumor
(Additional file 2: Figure S1)

## Slide 2
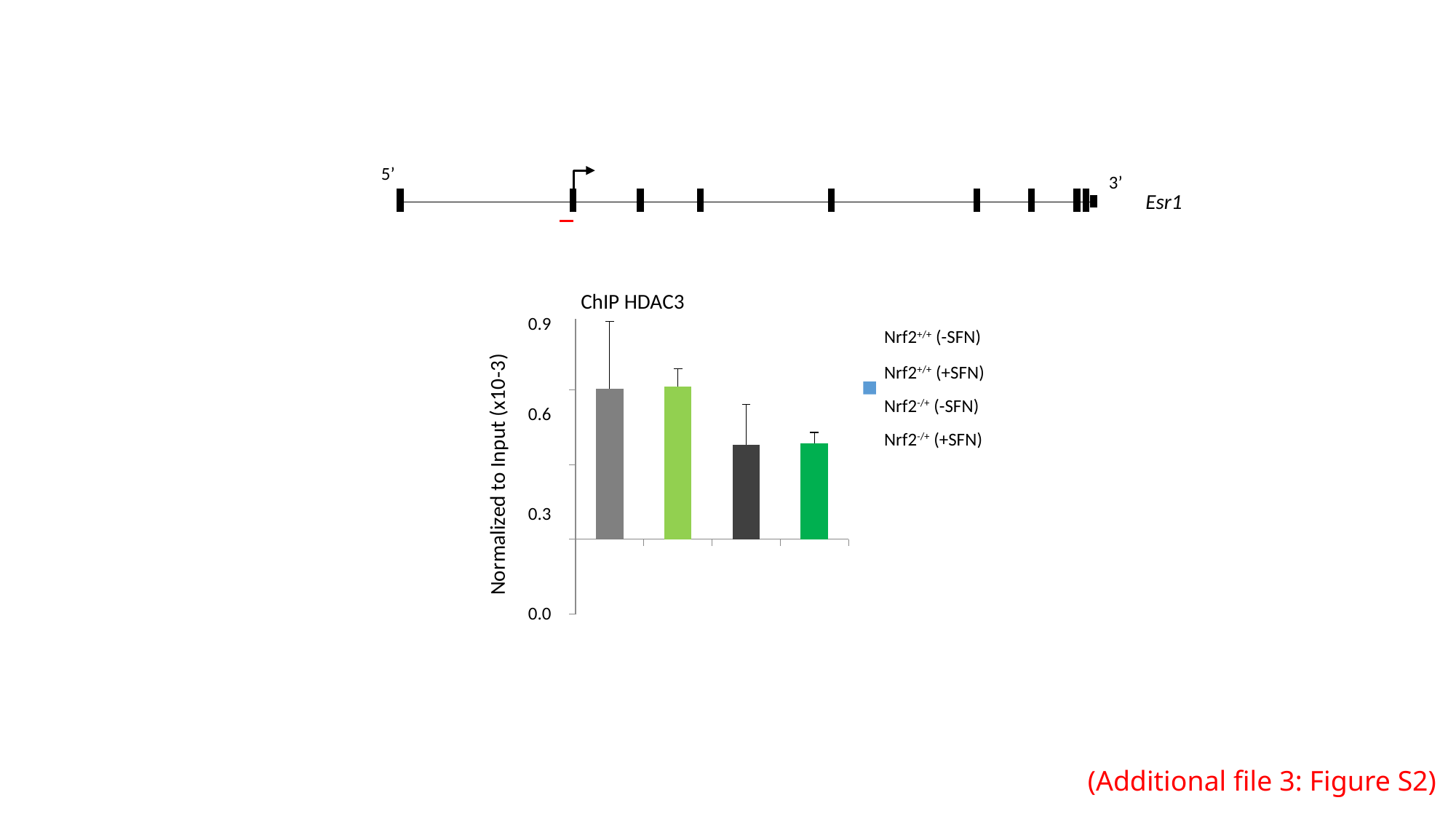

5’
3’
Esr1
ChIP HDAC3
### Chart
| Category | |
|---|---|
| Veh | 0.0006045977011494289 |
| SFN | 0.0006122950819672133 |
| Veh | 0.0003799472295514522 |
| SFN | 0.00038432432432432447 |0.9
Nrf2+/+ (-SFN)
Nrf2+/+ (+SFN)
Nrf2-/+ (-SFN)
0.6
Nrf2-/+ (+SFN)
0.3
0.0
(Additional file 3: Figure S2)

## Slide 3
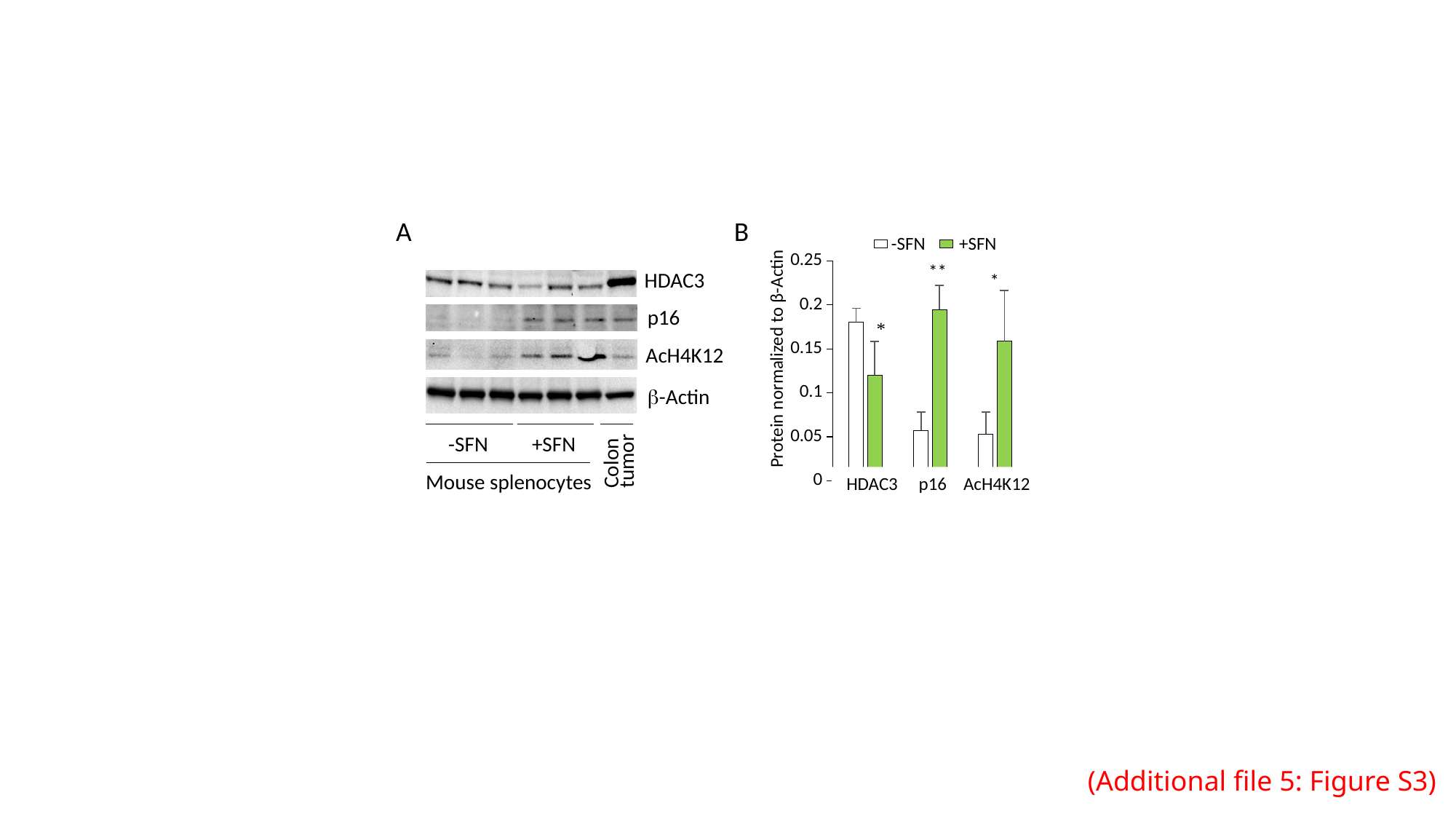

A
B
-SFN
+SFN
### Chart
| Category | | |
|---|---|---|HDAC3
p16
AcH4K12
Protein normalized to β-Actin
b-Actin
-SFN
+SFN
Colon
tumor
Mouse splenocytes
 HDAC3 p16 AcH4K12
(Additional file 5: Figure S3)

## Slide 4
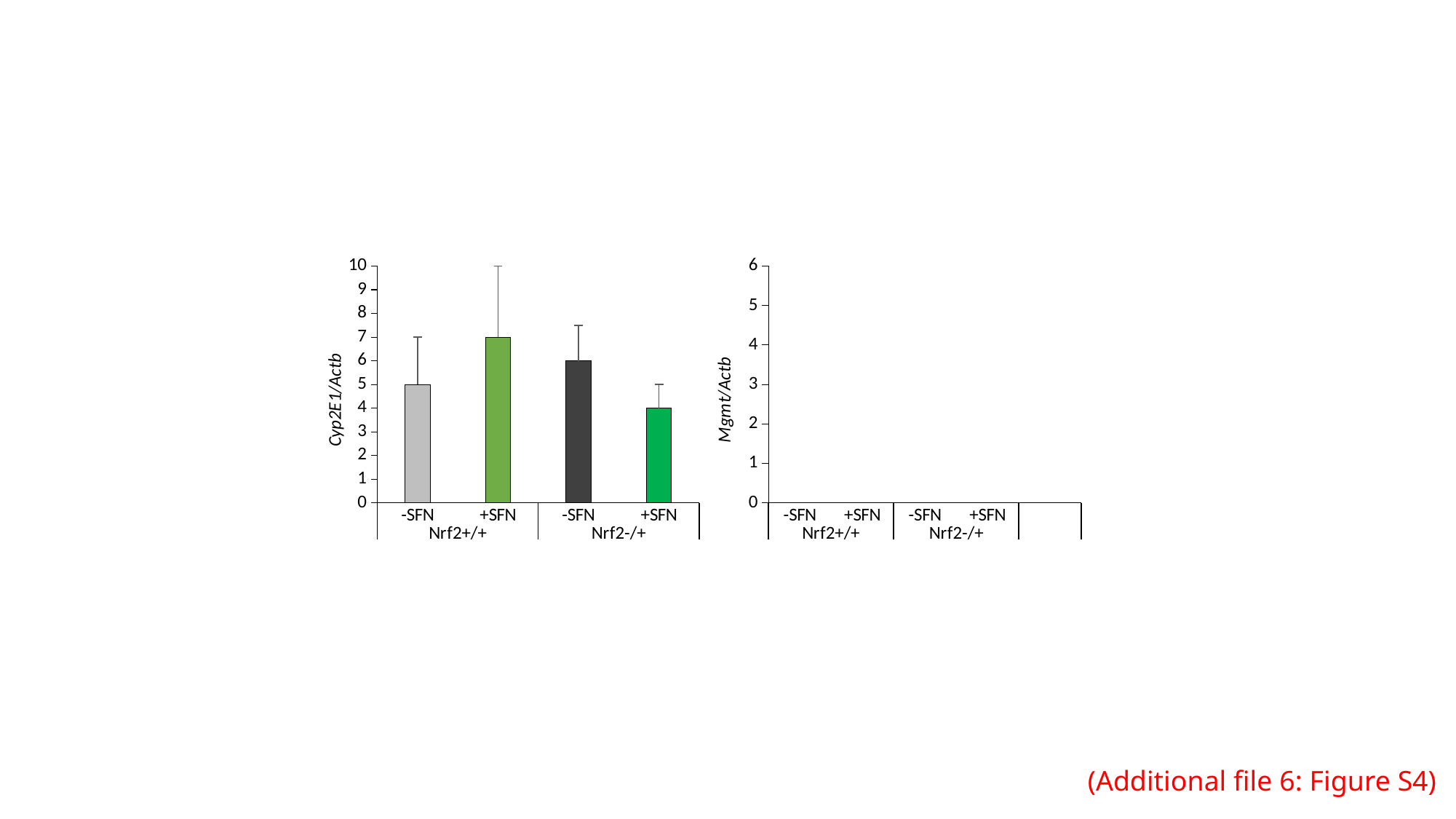

### Chart
| Category | Cyp2E1 |
|---|---|
| -SFN | 5.0 |
| +SFN | 7.0 |
| -SFN | 6.0 |
| +SFN | 4.0 |
### Chart
| Category | MGMT |
|---|---|
| -SFN | 2.0 |
| +SFN | 1.5 |
| -SFN | 2.5 |
| +SFN | 1.9 |(Additional file 6: Figure S4)

## Slide 5
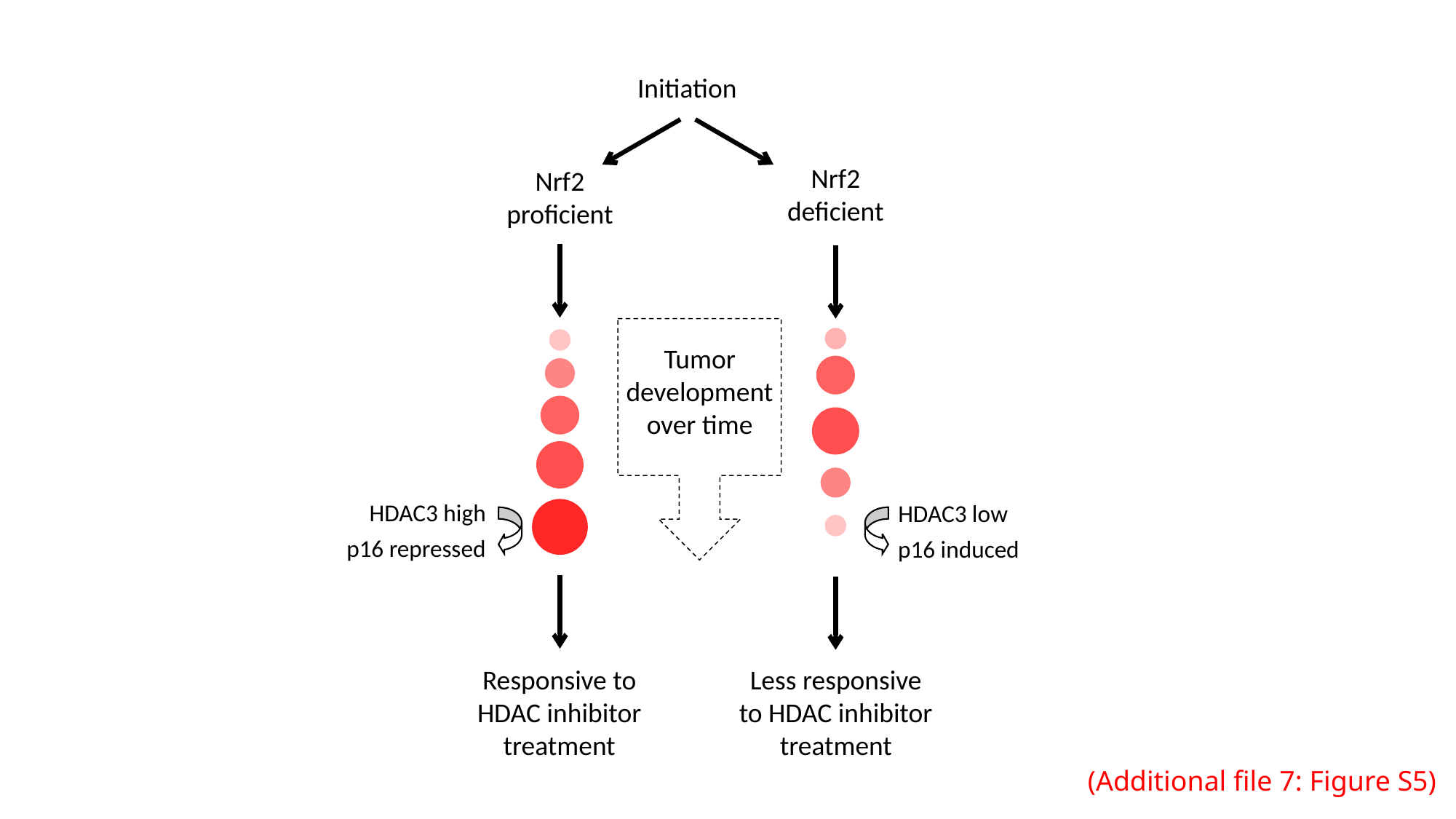

Initiation
Nrf2 deficient
Nrf2 proficient
Tumor development over time
HDAC3 high
HDAC3 low
p16 repressed
p16 induced
Responsive to HDAC inhibitor treatment
Less responsive to HDAC inhibitor treatment
(Additional file 7: Figure S5)
