# Supplementary material for: Nrf2 status affects tumor growth, HDAC3 gene promoter associations, and the response to sulforaphane in the colon
Source: Clin Epigenetics. 2015 Sep 18;7(1):102. doi: 10.1186/s13148-015-0132-y (PMC4575421; doi:10.1186/s13148-015-0132-y)
Supplement: Additional file 3: Table S2. — SFN and its metabolites in human plasma following ingestion of a BSE supplement. The levels of "SFN and its metabolites" in human plasma was measured using Liquid Chromatrography-Mass Spectrometry (LC-MS) methods, as described previously [27]. Calculated pharmacokinetic parameters - Tmax, Cmax, AUC, and half-life (mean) values are shown in 'rows' for each SFN metabolite, measured on Days 1 and 7, shown in respective columns. (DOCX 19 kb) [file 13148_2015_132_MOESM3_ESM.docx]

Additional file 4: Table S2

| **Pharmacokinetic**  **Parameters** | **SFN** | | **SFN-Cys** | | **SFN-CG** | | **SFN-GSH** | | **SFN-NAC** | |
| --- | --- | --- | --- | --- | --- | --- | --- | --- | --- | --- |
|  | **Day 1** | **Day 7** | **Day 1** | **Day 7** | **Day 1** | **Day 7** | **Day 1** | **Day 7** | **Day 1** | **Day 7** |
| Tmax (h) | 3.0 | 3.0 | 3.0 | 3.0 | 3.0 | 3.0 | 3.0 | 3.0 | 3.0 | 3.0 |
| Cmax (µM) | 0.027 | 0.022 | 0.148 | 0.125 | 0.389 | 0.145 | 0.274 | 0.210 | 0.084 | 0.075 |
| AUC (µM.h/L) | 0.075 | 0.080 | 0.453 | 0.393 | 1.281 | 0.579 | 0.835 | 0.646 | 0.278 | 0.274 |
| Half-life (h) | 1.18 | 2.27 | 2.09 | 1.57 | 2.23 | 3.42 | 1.45 | 1.04 | 3.48 | 3.80 |
